# Evaluation of TB Case Finding through Systematic Contact Investigation, Chhattisgarh, India

**DOI:** 10.1155/2015/670167

**Published:** 2015-07-06

**Authors:** Kshitij Khaparde, Pawan Jethani, Puneet K. Dewan, Sreenivas A. Nair, Madhav Rao Deshpande, Srinath Satyanarayana, Shamim Mannan, Patrick K. Moonan

**Affiliations:** ^1^World Health Organization, Country Office for India, New Delhi 110011, India; ^2^District TB Office, Rajnandgaon, Chhattisgarh 491441, India; ^3^Bill and Melinda Gates Foundation, New Delhi 110015, India; ^4^The Union, South-East Asia Regional Office, New Delhi 110016, India; ^5^Division of Tuberculosis Elimination, U.S. Centers for Disease Control and Prevention, Atlanta, GA 30329-4027, USA

## Abstract

*Rationale*. Contact investigation is an established tool for early case detection of tuberculosis (TB). In India, contact investigation is not often conducted, despite national policy, and the yield of contact investigation is not well described.* Objective*. To determine the yield of evaluating household contacts of sputum smear-positive TB cases in Rajnandgaon district, Chhattisgarh, India.* Methods*. Among 14 public health care facilities with sputum smear microscopy services, home visits were conducted to identify household contacts of all registered sputum smear-positive TB cases. We used a standardized protocol to screen for clinical symptoms suggestive of active TB with additional referral for chest radiograph and sputa collection.* Results*. From December 2010 to May 2011, 1,556 household contacts of 312 sputum smear-positive TB cases were identified, of which 148 (9.5%) were symptomatic. Among these, 109 (73.6%) were evaluated by sputum examination resulting in 11 cases (10.1%) of sputum smear-positive TB and 4 cases (3.6%) of smear-negative TB. Household visits contributed additional 63% TB cases compared to passive case detection alone.* Conclusion*. A standard procedure for conducting household contact investigation identified additional TB cases in the community and offered an opportunity to initiate isoniazid chemoprophylaxis among children.

## 1. Introduction

Contact investigation has been recommended internationally as a promising approach for identifying persons at high risk for developing tuberculosis (TB) and offers an opportunity for early case detection [1, 2]. However, in many low-resource, high-TB burden countries, contact investigation is not often conducted despite national policy [3, 4]. The Revised National Tuberculosis Control Programme of India (RNTCP) guidelines recommend contact investigation among household contacts of sputum smear-positive TB cases, providing isoniazid chemoprophylaxis for children less than 6 years of age who are in close contact of a sputum smear-positive case and are not diagnosed with TB [5, 6].

Studies have shown that active case finding among household contacts yields substantially more TB cases than passive case detection [7–10]. Contact investigations detect as many as 2.3% patients with pulmonary TB amongst close contacts in low-income and middle-income countries [3, 11]. In China, with a similar high-burden of TB in India, the yield for active TB case finding through contact investigation ranged from 0 to 6.9% in household contacts [12, 13].

Contact investigation also offers the opportunity to prevent the progression to active disease among persons with latent TB infection (LTBI). Studies from districts of south India concluded that there is suboptimal implementation of contact screening and isoniazid prophylaxis implementation under routine programmatic conditions [14, 15].

This study sought to evaluate the yield of screening household contacts of sputum smear-positive TB cases for TB by implementing a standard operational procedure for contact investigation using the existing health care staff and resources.

## 2. Methodology

### 2.1. Study Design and Setting

A community-based, cross-sectional, implementation research study was conducted in the largely rural and socioeconomically-depressed district of Rajnandgaon, in Chhattisgarh state, India—a population of 1.5 million. The district has implemented the DOTS strategy for 9 years, and has 52 public primary health care facilities, including 14 facilities where sputum smear microscopy services are offered.

### 2.2. Study Population

The Study population was all sputum smear-positive TB cases diagnosed from December 2010 to May 2011 and all household contacts identified through field-based contact investigation.

### 2.3. Intervention

A standard contact investigation protocol was developed which included a contact investigation checklist to be entered by a Health Care Worker (HCW) in local language. One HCW in each of all the 14 public health care facilities with sputum microscopy services was trained. Procedures specified that HCW was to conduct home visits (within 3 business days) for all sputum smear-positive TB cases notified, identify, and screen all household contacts for persons with any clinical symptoms consistent with tuberculosis as per the Standards for TB Care in India [16] (with the exception of including a cough of any duration (as opposed to cough of >2 weeks) to increase the sensitivity to find presumptive TB cases). All children, <6 years old, were referred for further clinical evaluation to assess eligibility for isoniazid chemoprophylaxis or treatment for TB disease.

The protocol specified the following actions. First, all households were to be visited and household contacts were to be recorded on a standard form. Second, symptomatic household contacts were provided with a contact slip (with unique identifier) and counseled to present for evaluation at the nearest health facility. Third, a second home visit was made to identify contacts missed in the first visit. Fourth, in case of any missed contacts, the contact slip was left with instructions for followup and appropriate evaluation at the closest health care facility. Fifth and lastly, one-time telephonic reminders were made after 1 week for contacts who failed to report for evaluation.

To monitor case finding outcomes among symptomatic household contacts, the unique identifier from the contact slip was entered in the laboratory register. The laboratory supervisor and the study principal investigator systematically monitored this intervention by revisiting the houses of at least 20% and 10% of the index cases, respectively. No additional costs were incurred beyond the general health system resources.

### 2.4. Case Definitions

Smear-positive pulmonary TB was defined as a patient with at least one sputum smear-positive result for acid-fast bacilli (AFB) on direct microscopy.

An index case was defined as the first sputum smear-positive case identified within a household. A household contact was defined as a person who lives with or shares a common kitchen with an index case.

Household contacts with pulmonary symptoms, who were negative for AFB in both samples, were prescribed a course of broad spectrum antibiotic for duration of 10–14 days. If symptoms persisted beyond 14 days of treatment, repeated sputum smear examinations (2 samples) were done. In repeated sputum examinations, patients with at least one sputum smear-positive results were diagnosed by the physician as having smear-positive pulmonary TB. Tuberculosis was suspected among contacts who presented symptoms of prolonged/unexplained fever and/or cough for more than 2 weeks, and/or unexplained weight loss (or no weight gain or history of failure to thrive among children). History of contact with a suspected or diagnosed case of smear-positive pulmonary TB within the last 2 years reinforced the suspicion of tuberculosis. The diagnosis was further based on sputum examination wherever possible, chest radiography examination, and Mantoux test (tuberculin skin test) according to the national guidelines [16]. The index case was advised to bring all children to the health centre for clinical TB evaluation by a medical officer. Those diagnosed with TB were treated according to the national guidelines [16]. Children that were found eligible for chemoprophylaxis (after ruling out TB) were administered preventive chemotherapy with isoniazid 5 mg/kg body weight daily for 6 months.

### 2.5. Data Collection and Analysis

Symptomatic contacts were matched with laboratory registers using the unique ID number from the contact slips. Household contact investigation forms were entered and analyzed in Epi-Info Version 3.5.1. Simple frequencies and percentages described the number and proportion of household contacts identified, household contacts evaluated, smear-positive pulmonary TB suspects among household contacts, household contacts with different types of TB, children <6 years of age diagnosed as having TB, and children <6 years of age not diagnosed as having TB and prescribed isoniazid chemoprophylaxis.

### 2.6. Ethical Considerations

The study was approved by the Ethical Committee of National Tuberculosis Institute, Bangalore, and by the Ethical Advisory Group of The Union. Local administrative approvals were also taken to conduct the study. No informed consent was required as the study offers minimal risk to the participants, and protocol activities were considered part of routine programmatic functions of TB treatment and care.

## 3. Results

**Figure 1:**
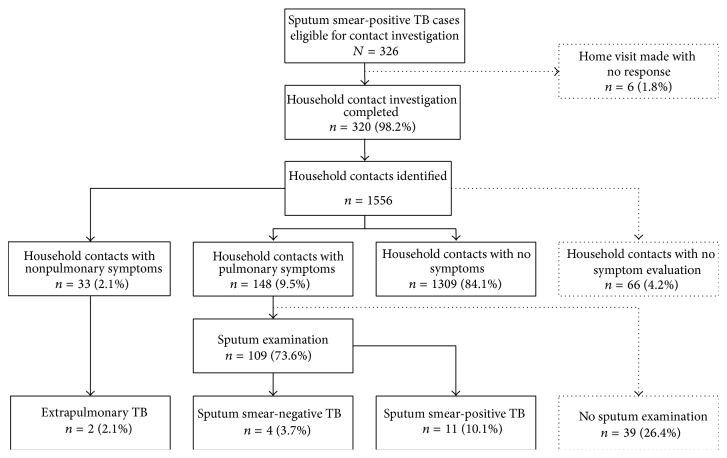
Flow diagram depicting the outcome of household contact investigation, Rajnandgaon district of the state of Chhattisgarh, India, from December 2010 to May 2011. Dotted boxes indicate potential missed opportunities for early TB case detection.

Of the 326 eligible index cases, for 6 (1.8%) we were unable to assess the number of household contacts (e.g., houses were locked at time of visits). Among the 320 contact investigations, 8 (2.5%) cases did not have any household contacts. In total, 1,556 household contacts were identified, with an average of 5 contacts per index case. No evaluations were completed for 66 (4.2%) contacts, even after a minimum of 2 visits and telephonic reminder. The median age of household contacts was 22 years (interquartile range: 11–39 years) with more than two-thirds of the contacts being >15 years of age. Home visits revealed 148 (9.5%) contacts who complained of cough, of which the majority (135 (91.2%)) were adults (Table 1). Laboratory registers showed that 109 (73.6%) symptomatic contacts underwent sputum examination, among whom 11 (10.1%) had sputum smear-positive results. Additional 4 contacts were subsequently diagnosed with sputum-negative TB, and 2 contacts were with extrapulmonary TB (Figure 1). Among the 312 index cases for whom contact investigations were conducted, 27 (8.7%) had at least one household contact diagnosed with TB. Overall, 27 cases of TB were diagnosed among household contacts, of which 10 cases were the result of passive case detection through self-reporting to the health system prior to the implementation of this intervention and 17 cases were the result of household visits, thus attributing additional 63% TB cases detected.

Among the 233 household contacts aged less than 6 years, 152 (65.2%) were successfully evaluated for TB, of which no TB cases were identified, and 148 (63.5%) were initiated on isoniazid chemoprophylaxis.

## 4. Discussion

We found, on average, 1 TB case for every 11 household contacts screened in a rural district of Chhattisgarh, India. A standardized protocol and systematic implementation of household contact investigation yielded an additional 17 TB cases of all types amongst the household contacts. This finding is similar to a study which concluded that standardized protocols allowed more effective monitoring of contact investigation and lead to more efficient and effective procedures [17].

The added yield may be an underestimate, as not all 148 contacts complaining of cough underwent sputum examination, even after repeated counseling. This exemplifies the challenges of implementing field-based contact investigations.

Similar to other studies in high-incidence, low-resource settings, we observed no pediatric cases among the household contacts, despite the higher risk of developing the disease [18]. However, these findings are not consistent with the studies for low-incidence, high-resource settings where identification of pediatric TB among household contacts was more common [19–21], suggesting that instances of pediatric TB may have been missed, which is not surprising, given the widely acknowledged challenges of diagnosis of pediatric TB in developing countries [22]. This study found that nearly two-thirds of the children less than 6 years of age were screened for TB and nearly all of these were initiated on isoniazid chemoprophylaxis, which is substantially better than the previously reported studies from India [14, 15].

The study had some limitations. Unfortunately, we did not implement a formal household census to determine household size or to track individual contacts through time. Moreover, a substantial portion (26.4%) of contacts with symptoms consistent with TB identified during the home visit did not present to RNTCP clinic for sputum collection. Thus, these individuals, and an unspecified number of unnamed contacts, may have self-reported to a private health care facility outside of the national TB programme and thus not counted as part of this intervention. We did not ascertain the reasons for failure to present nor do we know the final disposition of these persons. Further, some household contacts with symptoms may not have returned with the contact slip, leading to underascertainment of contacts examined and possible TB cases detected. It is possible that contacts developed TB disease after the home visits. Progression to TB is variable and we did not screen for latent tuberculosis infection in adults, and thus it is not known how much earlier these cases were diagnosed nor do we know how many would have been diagnosed with TB at later time.

This was one of the few studies in India that evaluated implementation of contact investigation without additional resources (i.e., using only HCW's of the existing general health system with no additional costs incurred in standardizing the procedures except for routine programme-provided training, printing, and travel costs), and indicates the feasibility of contact investigation and the potential contribution towards the national objective of early and improved TB case detection. Larger-scale implementation is needed to determine the generalizability of the findings.

## 5. Conclusion and Recommendations

Through systematic and active case finding among household contacts, we found that making home visits found additional TB cases beyond passive contact screening (waiting for contacts to develop symptoms and present on their own) demonstrating a promising approach towards improving access to early diagnosis of TB. Despite the operational difficulties, the management of contact investigation could vastly be improved by better implementation of the existing guidelines. We identified potential missed opportunities to bring persons at risk for TB to care. One in four contacts with symptoms consistent with TB failed to present to the RNTCP for sputum-smear microscopy. These individuals should be considered a high-priority for followup. Standardizing the contact investigation procedures will facilitate regular monitoring of this activity by the supervisors and programme managers. This will also facilitate the effective implementation of chemoprophylaxis to eligible children. Moreover, incorporating contact investigation information into routine national surveillance activities would further improve programme performance at the district, state, and national levels and elevate India's contribution to the global End TB Strategy [23, 24].

## Figures and Tables

**Table 1:** Age and sex distribution and clinical disposition of household contacts of sputum smear-positive TB, Rajnandgaon district of the state of Chhattisgarh, India, from December 2010 to May 2011.

Characteristic	No symptoms *n* (%)	Pulmonary symptoms (pulmonary TB suspects) *n* (%)	Nonpulmonary symptoms *n* (%)	TB cases detected directly from contact tracing efforts *n* (%)^1^	TB cases from passive detection^2^ *n* ^3^	Total
Age in years						
0–6	226 (16.4)	2 (1.4)	5 (15.2)	0 (—)	0	233
7–14	255 (18.6)	11 (7.4)	4 (12.1)	3 (20.0)	0	270
≥15	894 (65.0)	135 (91.2)	24 (72.7)	14 (8.8)	10	1,053
Sex						
Male	640 (46.5)	62 (41.9)	12 (36.4)	6 (8.1)	4	714
Female	735 (53.5)	86 (58.1)	21 (63.6)	11 (10.3)	6	842
Total	1,375	148	33	17	10	1,556

^1^Percent among presumptive TB cases per category row.

^2^Household contacts who self-reported to the health system prior to contact tracing.

^3^Not included in row total.
